# Expanding the Menu: Are Polyphagy and Gene Family Expansions Linked across Lepidoptera?

**DOI:** 10.1093/gbe/evab283

**Published:** 2021-12-24

**Authors:** Thijmen Breeschoten, Corné F H van der Linden, Vera I D Ros, M Eric Schranz, Sabrina Simon

**Affiliations:** 1 Biosystematics Group, Wageningen University & Research, The Netherlands; 2 Laboratory of Virology, Wageningen University & Research, The Netherlands

**Keywords:** gene family evolution, gene family expansion, herbivory, butterfly–plant interactions, Lepidoptera

## Abstract

Evolutionary expansions and contractions of gene families are often correlated with key innovations and/or ecological characteristics. In butterflies and moths (Lepidoptera), expansions of gene families involved in detoxification of plant specialized metabolites are hypothesized to facilitate a polyphagous feeding style. However, analyses supporting this hypothesis are mostly based on a limited number of lepidopteran species. We applied a phylogenomics approach, using 37 lepidopteran genomes, to analyze if gene family evolution (gene gain and loss) is associated with the evolution of polyphagy. Specifically, we compared gene counts and evolutionary gene gain and loss rates of gene families involved in adaptations with plant feeding. We correlated gene evolution to host plant family range (phylogenetic diversity) and specialized metabolite content of plant families (functional metabolite diversity). We found a higher rate for gene loss than gene gain in Lepidoptera, a potential consequence of genomic rearrangements and deletions after (potentially small-scale) duplication events. Gene family expansions and contractions varied across lepidopteran families, and were associated to host plant use and specialization levels. Within the family Noctuidae, a higher expansion rate for gene families involved in detoxification can be related to the large number of polyphagous species. However, gene family expansions are observed in both polyphagous and monophagous lepidopteran species and thus seem to be species-specific in the taxa sampled. Nevertheless, a significant positive correlation of gene counts of the carboxyl- and choline esterase and glutathione-S-transferase detoxification gene families with the level of polyphagy was identified across Lepidoptera.

Significance

Major expansions of gene families involved in plant feeding, such as detoxification of plant specialized metabolites, are hypothesized to facilitate and enable polyphagy in herbivorous insects. To test this hypothesis, we apply a comparative phylogenomics framework. We find gene family expansions to occur in both monophagous and polyphagous Lepidoptera, and a significant positive correlation between the size of the detoxification gene families *CCE* and *GST* with level of polyphagy. Thus, gene family sizes are variable across monophagous and polyphagous Lepidoptera but expansions can be correlated to host plant breadth for specific gene families.

## Introduction

The insect order Lepidoptera (butterflies and moths) form an insect super radiation with ∼160,000 described species found in nearly all ecosystems on earth (Grimaldi and Engel 2005; Pogue 2009; van Nieukerken et al. 2011). Furthermore, Lepidoptera are one of the largest radiations of plant-feeding insects, with plant feeding having evolved from inner plant tissue (via concealed external feeding) to exposed folivory (Menken et al. 2010; Mitter et al. 2017). The diversity of Lepidoptera and the expansion of the order have been linked to the close association with- and rise of angiosperms (Ehrlich and Raven 1964; Mitter et al. 2017; Allio et al. 2021). The coevolutionary relationship through a process termed “escape-and-radiate” has long been hypothesized to be the driving force for the diversification of both flowering plants and butterflies (Ehrlich and Raven 1964; Thompson 1989). However, Lepidoptera primarily feed upon plant families that radiated before most butterfly and moth families did (Labandeira and Sepkoski 1993; Janz and Nylin 1998). Therefore, studies argue that host-shifts, through colonization and specialization, have likely shaped the patterns of insect–plant associations (Jermy 1984; Janz and Nylin 1998; Braby and Trueman 2006). The study of the close interactions and adaptations of Lepidoptera to their host plants have benefited from genomic analysis (Simon et al. 2015; Triant et al. 2018; Birnbaum and Abbot 2020).

Genomic changes can be correlated to adaptive changes and ecological characteristics associated to plant feeding (Edger et al. 2015; Simon et al. 2015; Gloss et al. 2019). Correlating genomic changes to evolutionary processes, like radiation events and dietary shifts, can be an important step to reveal the genomic drivers of these processes (Seppey et al. 2019; Allio et al. 2021). Genomic changes vary from point mutations, to expansions of specific gene-families up to genome duplications. Duplicated gene copies can lead to a selective advantage and may eventually be preserved by selective forces, or alternatively be nonbeneficial and thus lost (Innan and Kondrashov 2010). The selective advantage of duplicated genes can be due to increased gene dosage and/or gene neofunctionalization. For example, duplicated and neofunctionalized genes might facilitate the detoxification of novel plant defense toxins and thus potentially expand the breadth of accepted host plant species (Wen et al. 2006; Heidel-Fischer et al. 2019).

Host specialization, on a single or few host plant species within one plant family (described as monophagy within this study), is most common among herbivorous insects. Whereas some herbivorous insects, including some of the most devastating pest species, are polyphagous meaning they are able to feed on a variety of plant species belonging to different families (Schoonhoven et al. 2005; Voelckel and Jander 2014). Polyphagous species likely evolved and maintained detoxification mechanisms with a broad substrate specificity as a counter-response to the large variety of plant defense toxins, or specialized metabolites, they encounter (Heidel-Fischer and Vogel 2015).

A general insect detoxification mechanism occurs via the three-step detoxification pathway for which a series of generally recognized gene families are involved (Brattsten 1988; Kant et al. 2015). In the first step, P450 monooxygenases (*P450*s) and carboxyl- and choline esterases (*CCE*s) make the plant toxin more hydrophilic. During the second step, UDP-glycosyltransferases (*UGT*s) and glutathione-S-transferases (*GST*s) conjugate the compounds to endogenous molecules increasing the polarity and hydrophilicity of the molecules even further, after which in the final third step membrane transporters like ATP-binding cassettes (*ABC*s) move the compounds for excretion (Feyereisen 1999; Voelckel and Jander 2014; Heidel-Fischer and Vogel 2015; Kant et al. 2015; Dermauw et al. 2020). Although monophagous species have often evolved specialized gene functions to target specific host plant defenses (Ratzka et al. 2002; Wittstock et al. 2004; Wheat et al. 2007; Fischer et al. 2008; Heidel-Fischer et al. 2019), polyphagy has often been associated to gene family expansions.

Genome studies of polyphagous arthropods show the occurrence of major gene family expansions of detoxification and digestion related families, for example, in Lepidoptera (Xu et al. 2016), Hemiptera (Chen et al. 2016), and Acari (Dermauw et al. 2013). In the fall armyworm, *Spodoptera frugiperda*, observed expansions were primarily due to tandem duplications forming a suggested adaptation mechanism to enable polyphagy (Gouin et al. 2017). Similarly, gene expansions have been linked to polyphagy for the cotton leafworm, *Spodoptera litura* (Cheng et al. 2017).

Gene family expansions are hypothesized to be causal for the emergence of polyphagy in Lepidoptera (Cheng et al. 2017; Gouin et al. 2017). Specific gene copies and functional diversity of Clan 3 *P450* subfamilies have been linked to diet complexity in multiple Lepidoptera (Calla et al. 2017). This indicated a correlation between gene family dynamics (e.g., duplication and functionalization level) and xenobiotic metabolism (Calla et al. 2017). Nonetheless, in a larger comparative study a relationship between detoxification gene family sizes (*P450*, *CCE*, and *GST*) and feeding preference was found in multiple insect orders but not in the studied seven Lepidoptera species (Rane et al. 2016). However, studies on the association between gene family expansions and herbivory among a larger number of lepidopteran species has not yet been reported.

In this study, we applied a comparative phylogenomic approach using available high-quality lepidopteran genomes (37) spanning the lepidopteran tree of life in order to examine the evolution of gene family expansions associated with host plant use. The lepidopteran species vary in many characteristics such as feeding habit, host plant species range, specialized metabolite acceptance, and pest status. Therefore, we examined the correlation between these herbivory-characteristics and genomic changes.

Gene family evolution, in terms of gene gain and loss rates, differed across lepidopteran families and was associated to ecological and evolutionary characteristics. Gene family expansions occur in both polyphagous and monophagous species. However, we found a significant positive correlation between the size of the detoxification gene families *CCE* and *GST* with levels of polyphagy. In summary, detoxification gene family sizes are variable across monophagous and polyphagous Lepidoptera but expansions can be correlated to level of polyphagy for specific gene families.

## Results

### Genomes, Gene Families, and Species Tree Reconstruction

**Fig. 1.**
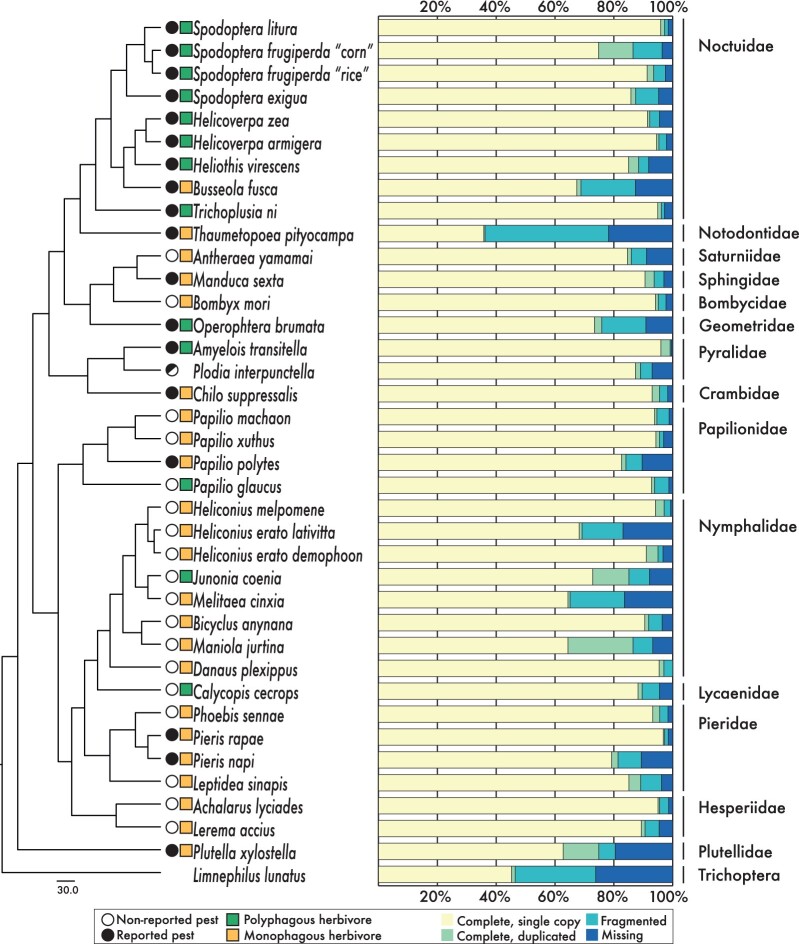
ML tree topology based on 1,367 single-copy BUSCOs from 37 lepidopteran genomes. Species pest status and feeding style are given, discriminating between monophagous and polyphagous species (supplementary table 11, Supplementary Material online). Feeding style is not provided for *Plodia interpunctella*, since this species feeds on dried products. For every species the taxonomic family is given (right). Stacked bar graphs present the BUSCO quality assessment of the genome gene sets used in this study.

**Fig. 2.**
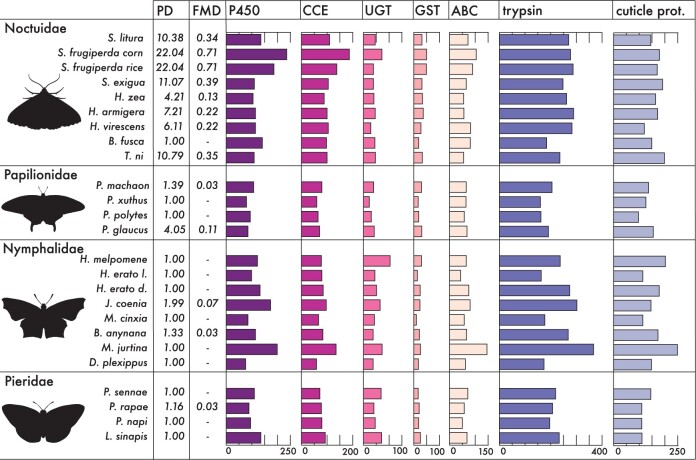
Graph showing the gene counts of seven gene families for four lepidopteran families: Noctuidae, Papilionidae, Nymphalidae, and Pieridae. Gene families include five families involved in metabolite detoxification: *P450*, P450 monooxygenase; *CCE*, carboxyl- and choline esterase; *UGT*, UDP-glycosyltransferase; *GST*, glutathione-S-transferase; *ABC*, ATP-binding cassette; one family involved in digestion: trypsin and one family putatively involved in protection of the insect midgut: insect cuticle protein. PD values represent the PD of the host plant families within each lepidopteran diet. The PD values are scaled, with 1 being monophagous and >1 polyphagous species. FMD values show the FMD of metabolites employed by the host plant families within the lepidopteran diets.

We analyzed 37 Lepidoptera genomes for which complete gene sets were available (on September, 2019) and included one outgroup represented by the sister clade Trichoptera. The average number of protein-coding sequences was 17,589 genes and ranged from 12,240 to 29,415 per species (supplementary table 1, Supplementary Material online and table 1). Based on benchmarking universal single-copy orthologs (BUSCO) analyses, the majority of species (85%) had a completeness of >75% with an average completeness of 86.8% (fig. 1). The number of functionally annotated protein sequences from InterProScan ranged from 10,723 to 32,131 (supplementary table 2, Supplementary Material online) and from BlastP against the UniRef50 database from 13,279 to 40,328 (supplementary table 3, Supplementary Material online). We calculated the gene number of various herbivory related gene families (*P450*s, *CCE*s, *UGT*s, *GST*s, *ABC*s, trypsins, and insect cuticle proteins; fig. 2; supplementary table 4, Supplementary Material online) based on InterProScan and Uniref50 identifiers (supplementary table 5, Supplementary Material online).

**Table 1:** Overview of Predicted Genes of Four Major Lepidopteran Families (Noctuidae, Papilionidae, Nymphalidae, and Pieridae)

Family	Species	Predicted Genes	Annotated Detoxification Genes	% Detox. Genes	Annotated Detoxification, Trypsin and Cuticle Genes	% Detox., Trypsin and Cuticle Genes
Noctuidae	*Spodoptera litura*	15317	395	2.58	810	5.29
	*Spodoptera frugiperda* “corn”	21779	649	2.98	1,105	5.07
	*Spodoptera frugiperda* “rice”	26356	502	1.90	959	3.64
	*Spodoptera exigua*	18477	351	1.90	790	4.28
	*Helicoverpa zea*	15128	324	2.14	750	4.96
	*Helicoverpa armigera*	17082	353	2.07	814	4.77
	*Heliothis virescens*	15099	358	2.37	761	5.04
	*Busseola fusca*	19417	384	1.98	717	3.69
	*Trichoplusia ni*	14384	344	2.39	778	5.41
Average per family			406.67	2.26	831.56	4.68
Papilionidae	*Papilio machaon*	15497	322	2.08	663	4.28
	*Papilio xuthus*	13102	243	1.85	529	4.04
	*Papilio polytes*	12244	266	2.17	525	4.29
	*Papilio glaucus*	15692	297	1.89	643	4.10
Average per family			282.00	2.00	590.00	4.18
Nymphalidae	*Heliconius melpomene*	20075	399	1.99	838	4.17
	*Heliconius erato lativitta*	14613	281	1.92	558	3.82
	*Heliconius erato demophoon*	14517	368	2.53	820	5.65
	*Junonia coenia*	19234	439	2.28	887	4.61
	*Melitaea cinxia*	16667	262	1.57	553	3.32
	*Bicyclus anynana*	22642	324	1.43	765	3.38
	*Maniola jurtina*	36294	579	1.60	1,195	3.29
	*Danaus plexippus*	15130	253	1.67	575	3.80
Average per family			363.13	1.87	773.88	4.01
Pieridae	*Phoebis sennae*	16493	339	2.06	703	4.26
	*Pieris rapae*	13188	286	2.17	603	4.57
	*Pieris napi*	13622	290	2.13	596	4.38
	*Leptidea sinapis*	18049	393	2.18	737	4.08
Average per family			327.00	2.13	659.75	4.32

Note.—Number of predicted genes based on the genome annotations; number of annotated detoxification genes (from families *P450*, *CCE*, *UGT*, *GST*, and *ABC*); the number of annotated detoxification genes as percentage of the predicted genes; number of annotated detoxification, trypsin, and insect cuticle genes; and the number of annotated detoxification, trypsin, and insect cuticle genes as percentage of the predicted genes are listed in this table. Further, the averages for each Lepidoptera family are given.

OrthoFinder identified 21,610 orthologous groups (OGs) (supplementary table 6, Supplementary Material online; see supplementary table 6*B*, Supplementary Material online, for the OGs and associated Pfam, InterProScan, and UniRef50 annotations). These resulting orthologous protein groups and the corresponding gene count data sets (supplementary table 7, Supplementary Material online) were used as input for the CAFE analyses (Computational Analysis of gene Family Evolution) in CAFE v. 4.2.1 (Hahn et al. 2005; De Bie et al. 2006), after filtering for high variance groups. We performed CAFE analyses for several data sets. The “all gene families data set” consisted of 21,148 OGs (supplementary table 8, Supplementary Material online) and the “5 gene families data set” consisted of 574 OGs (supplementary table 9, Supplementary Material online), including only OGs belonging to five specific gene families involved in specialized metabolite detoxification, namely *P450*s, *CCE*s, *UGT*s, *GST*s, and *ABC*s. The “single gene family data sets” consisted of 197 OGs for the *P450* gene family, 148 OGs for *CCE*, 64 OGs for *UGT*, 32 OGs for *GST*, 154 OGs for *ABC*, 383 OGs for trypsin, and 203 OGs for the insect cuticle gene family (supplementary table 10, Supplementary Material online).

The species phylogeny was constructed using the protein sequences of 1,367 single-copy and complete BUSCO genes (fig. 1, left). The 50 independent maximum likelihood (ML) tree searches returned one unique tree topology. Our phylogeny contained six lepidopteran superfamilies of which four consisted of more than one species, and 14 families of which six consisted of more than one species. This resulted in a Lepidoptera-clade wide species representation which is consistent with the comprehensive phylogeny by Kawahara et al. (2019).

### Ecological Host Data and Diversity Metrics

Based on host plant family range per lepidopteran species (supplementary table 11, Supplementary Material online), we calculated a phylogenetic diversity (PD) index (supplementary table 12, Supplementary Material online and fig. 2). The scaled PDs ranged from 1, for monophagous species feeding on host(s) within a single plant family, to 22.04 for the major polyphagous species *S. frugiperda*, feeding on hosts from 74 different plant families.

Our data set of specialized metabolite content per host plant species consisted of 3,831 entries, and is based on ∼750 literature sources (supplementary table 13, Supplementary Material online; on plant family level). Further, based on the host plant acceptance range per lepidopteran species, we calculated a “functional metabolite diversity” (FMD) index (supplementary table 14, Supplementary Material online and fig. 2). The FMD as calculated for all polyphagous species ranged from 0.03 for *Papilio machaon*, able to metabolize the specialized metabolites from host plant species within Apiaceae and Rutaceae, to 0.71 for *S. frugiperda*.

### Gene Family Expansions and Contractions

We calculated the gene counts of the seven target gene families involved in plant feeding (*P450*, *CCE*, *UGT*, *GST*, *ABC*, trypsin, and insect cuticle) for all 38 genomes. The gene family sizes varied across the species with *P450* family ranging from 236 genes in *S. frugiperda* to 73 in *Chilo suppressalis*, *CCE* ranging from 187 in *S. frugiperda* to 59 in *Danaus plexippus*, *UGT* from 104 in *Heliconius melpomene* to 23 in *Papilio xuthus*, *GST* from 50 in *S. frugiperda* to 10 in *Melitaea cinxia*, *ABC* from 146 in *Maniola jurtina* to 43 in *Heliconius erato lativitta*, trypsin from 367 in *M. jurtina* to 140 in *Thaumetopoea pityocampa*, and finally the insect cuticle protein family with 249 genes in *M. jurtina* to 97 genes in *Papilio polytes* (supplementary table 4, Supplementary Material online)*.* We focused on the gene counts of these gene families within the four focal Lepidoptera families.

The butterfly families Noctuidae, Papilionidae, Nymphalidae, and Pieridae differed in herbivorous traits and range of polyphagy and each was represented by at least >2 species. The average number of total gene counts for the seven gene families was greatest for the Noctuidae (831.56; table 1 and fig. 2), in concordance with the widest range of accepted host plants (PD, ranging between 1 and 22.04, and FMD, ranging between 0.13 and 0.71; fig. 2). The Noctuidae also had the highest average number of genes when only the five detoxification gene families were included (406.67), with the second largest number of genes found for the Nymphalidae (773.88 and 363.13). However, a high gene count can be the result of an overall larger number of predicted genes dependent on the quality of the genome annotation. Therefore, we normalized the number of genes from the target gene families using the percentage of the total number of predicted genes (table 1). Again, the size of the gene families was highest in Noctuidae (4.68%) but the order of the second largest shifted to Pieridae (4.32%). However, the differences were small and disproportionate to the differences in PD and FMD values (fig. 2), which reflect the level of polyphagy.

**Fig. 3.**
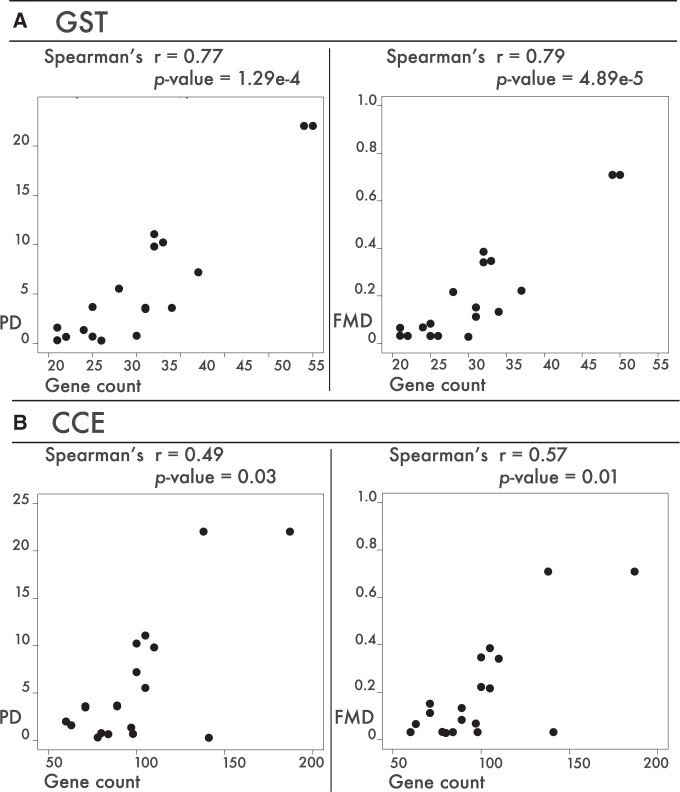
Scatterplots showing the distribution of gene counts of *GST* genes (*A*, *x* axes) or *CCE* genes (*B*, *x* axes) against the PD index values (*y* axes, left) or FMD index values (*y* axes, right) of all polyphagous Lepidoptera species. The Spearman correlation coefficient and *P*-value are given above each plot.

We calculated the correlation and level of significance between the PD values and gene counts of the seven target gene families across polyphagous Lepidoptera (supplementary table 15, Supplementary Material online). There was a significant positive correlation between gene counts of the detoxification gene families *CCE* (*r* = 0.49, *P*=0.03) and *GST* (*r* = 0.77, *P*=1.29e^−4^) in polyphagous species and the level of polyphagy as represented by the PD index (fig. 3*A*). The *GST* gene family was also significant positively correlated in the additional tests when only the single *S. frugiperda* rice strain was included (supplementary table 15, Supplementary Material online).

Further, we also calculated the correlation coefficient and level of significance between the FMD and gene counts of the seven target gene families (supplementary table 15, Supplementary Material online). Again, there was a significant positive correlation between the gene counts of gene families *CCE* (*r* = 0.57, *P*=0.01) and *GST* (*r* = 0.79, *P*=4.89e^−5^) in polyphagous species and the level of polyphagy as represented by the FMD (fig. 3*B*). Further, both *CCE* and *GST* gene families remained positively correlated when only the single *S. frugiperda* rice strain was included (supplementary table 15, Supplementary Material online).

Finally, in all cases (both for PD and FMD values), we tested for correlation significance of the seven gene families as fraction of the total number of annotated genes. The *GST* gene family was significant positively correlated for all analyses (supplementary table 15, Supplementary Material online).

### Gene Family Evolution

The analyses of gene family expansions and contractions using CAFE and inclusion of all gene families, using the “all gene families data set,” resulted in an overall rate of change, λ, of 0.0023 (likelihood score −641908; supplementary table 16, Supplementary Material online). Calculating a distinct rate for gene gain, λ = 0.0015 gain/gene/Myr, and gene loss, μ = 0.0032 loss/gene/Myr, resulted in a greater likelihood score (−628685; supplementary table 16, Supplementary Material online) and thus was preferred over calculating a single rate of change (Hahn et al. 2005).

**Fig. 4.**
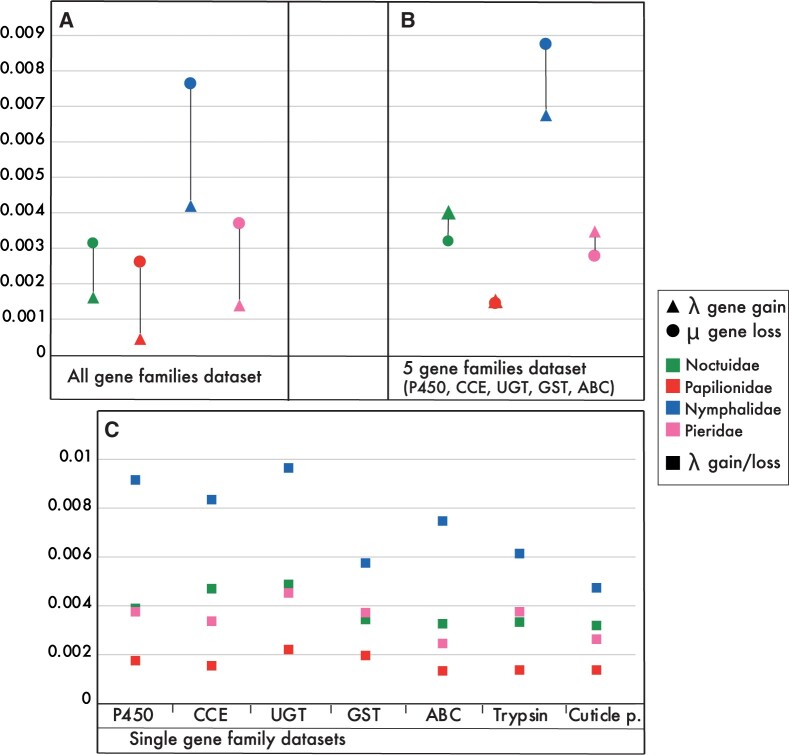
Estimates of gene family evolution rates as calculated with CAFE. The parameters are calculated for the four lepidopteran families Noctuidae, Papilionidae, Nymphalidae, and Pieridae. Rates for gene loss (circles, loss/gene/Myr, μ) and gene gain (triangles, gain/gene/Myr, λ) calculated for: (*A*) “all gene families data set”; and (*B*) “5 gene families data set,” which include the detoxification gene families P450 monooxygenase *(P450*), carboxyl- and choline esterase *(CCE*), UDP-glycosyltransferase *(UGT*), glutathione-S-transferase *(GST*), and ATP-binding cassette *(ABC*). Single rates of change (squares, either gain or loss/gene/Myr, λ) calculated for: (*C*) “single gene family data sets” of the five main detoxification gene families, and trypsin and insect cuticle protein families.

We associated gene expansion and contraction rates with the ecology and herbivorous characteristics for the four lepidopteran families, Noctuidae, Papilionidae, Nymphalidae, and Pieridae, separately. The λ (gain) and μ (loss) values calculated when all gene families were included, using the “all gene families data set,” showed a higher rate for gene loss for all butterfly families (fig. 4*A* and supplementary table 16, Supplementary Material online). Both λ and μ rates were highest for Nymphalidae compared with the other families, with the rate of gene loss (μ = 0.0076), almost twice as large as the highest second value (μ = 0.0036) for Pieridae (fig. 4*A*).

The gene gain and loss rates by inclusion of only the five detoxification gene families (*P450*, *CCE*, *UGT*, *GST*, and *ABC*), using the “5 gene families data set,” was again highest for Nymphalidae compared with the other families, with a higher rate for gene loss (λ = 0.0067, μ = 0.0087). Papilionidae had a similar rate for λ (0.0015) and μ (0.0014), whereas both Noctuidae (λ = 0.0040, μ = 0.0032) and Pieridae (λ = 0.0035, μ = 0.0028) showed a higher rate for gene gain over gene loss (fig. 4*B* and supplementary table 16, Supplementary Material online).

Finally, the single rate of change (λ) as calculated for each of the seven gene families (including the trypsin and cuticle protein families), using the “single gene family data sets,” differed across the Lepidoptera families. The calculated λ was consistently highest for the Nymphalidae (*P450* λ = 0.0091, *CCE* λ = 0.0083, *UGT* λ = 0.0096, *GST* λ = 0.0057, *ABC* λ = 0.0075, trypsin λ = 0.0061, insect cuticle λ = 0.0047), whereas Papilionidae (*P450* λ = 0.0017, *CCE* λ = 0.0015, *UGT* λ = 0.0022, *GST* λ = 0.002, *ABC* λ = 0.0013, trypsin λ = 0.0013, insect cuticle λ = 0.0014) had the lowest rate of change for all studied gene families. Both Pieridae (*P450* λ = 0.0037, *CCE* λ = 0.0033, *UGT* λ = 0.0045, *GST* λ = 0.0037, *ABC* λ = 0.0024, trypsin λ = 0.0037, insect cuticle λ = 0.0026) and Noctuidae (*P450* λ = 0.0038, *CCE* λ = 0.0047, *UGT* λ = 0.0048, *GST* λ = 0.0034, *ABC* λ = 0.0032, trypsin λ = 0.0033, insect cuticle λ = 0.0032) showed similar λs for most gene families but for *CCE*, *ABC*, and the insect cuticle protein family, the difference in rate of change was larger (fig. 4*C* and supplementary table 16, Supplementary Material online).

## Discussion

In this study, we evaluated if gene family expansions are correlated with polyphagy across Lepidoptera. We examined and associated genomic data of 37 lepidopteran genomes to the range of accepted host plants and their specialized metabolite contents. Specifically, we investigated gene family repertoires and expansion and contraction rates of gene families putatively involved in metabolite detoxification and digestion.

### Lepidopteran Phylogenomic Framework and Data Quality

Predictions on gene and genome evolution across a range of species depend on the robustness and accuracy of the species phylogeny. Our targeted phylogenetic reconstruction of lepidopteran species with completed genomes (fig. 1) was consistent with the comprehensive phylogeny by Kawahara et al. (2019). Further, the compared lepidopteran genomes should be of similar quality and completeness to avoid biases. The mean number of predicted proteins was 17,590 (SD = 4,785.73) which falls within the expected range of insect genomes (Waterhouse 2015). For a few species the number of reported predicted proteins was higher than the average. For example, 29,415 proteins in the pine processionary moth (*Thaumetopoea pityocampa*) (Gschloessl et al. 2018) and 36,294 predicted proteins in the meadow brown butterfly (*M. jurtina*) (Singh et al. 2020). However, this difference was reduced due to the selection of the 21,610 orthogroups, excluding ungrouped and unplaced sequences, specific subselections of particular gene families, and selection and focus on specific lepidopteran families.

Comparative genetics and genomics rely heavily on the results of previous studies by, for example, analyzing assembled data from various sources and laboratories using different analytical methods. Assembly and annotation quality might vary accordingly. Consequently, critically assessing the reliability of the data throughout the analyses is important. Therefore, we have performed various quality checks and additional analyses: 1) exclusion of suspicious data (e.g., assigning *M. jurtina* as an outlier in the analyses), 2) proteome completeness analyses of available genomes, 3) removing isoform duplications from the genomes, and 4) applying the error model for the gene family evolution analyses to account for annotation errors. The quality of genome assemblies and gene annotations are continuously improving with recent major improvements by inclusion of long-read sequencing (Hotaling et al. 2021). Consequently, the results and our conclusions which are based on limited data sets need retesting and revisiting using a denser taxon sampling and higher quality genome assemblies and gene predictions.

### Gene Evolution in Lepidoptera

Using our lepidopteran phylogenomic framework and inclusion of all gene families, we estimated an overall rate of change, λ, of 0.0023 (gains/losses/Myr). Our estimate was consistent with gene turnover estimates of other insect clades including *Drosophila* (λ = 0.0012; Hahn et al. 2007) and *Anopheles* (λ = 0.0031; Neafsey et al. 2015), and other taxa, such as yeast (λ = 0.002; Hahn et al. 2005) and mammals (λ = 0.0016; Demuth et al. 2006). When we calculated a separate value for gene gain and loss, the overall loss rate (μ = 0.0032) was higher than the gene gain rate (λ = 0.0015). This individual rate for gene gain (λ) was similar to the single estimated parameter for gene gain/loss calculated in Lepidoptera based on five genomes in a recent study (λ = 0.0014) (Thomas et al. 2020).

Both of our calculated turnover estimates were close to the general rates in other taxa but the difference in λ and μ are larger than in estimates of beetles, Coleoptera (λ = 0.0019, μ = 0.0018) (Seppey et al. 2019). This shows a higher rate of gene loss over gene gain within Lepidoptera. Indeed, gene loss can be seen as an important aspect in the evolution of species in terms of adaptive and/or neutral evolution (Albalat and Cañestro 2016).

It has been suggested that in the ancestry of Lepidoptera a large-scale genome duplication event occurred, before the radiation of Lepidoptera (<300 Ma) (Li et al. 2018). However, the occurrence of a whole-genome duplication event as hypothesized in lepidopteran ancestors has been questioned after reanalyses of the data (Nakatani and McLysaght 2019). Alternatively, small-scale gene duplications and segmental duplications by increased activity of transposable elements could explain the observed signs of duplication (Roelofs et al. 2020). Genome rearrangements and gene loss will gradually remove the signs of duplication events (Roelofs et al. 2020). Indeed, the genome size of extant Lepidoptera is similar to other insect orders (Hanrahan and Johnston 2011; Triant et al. 2018; Gregory 2020). Moreover, in a recent study on gene content evolution in Arthropoda, the common ancestor of Lepidoptera had the highest number of emergent gene families in comparison to all other insect clades (Thomas et al. 2020). Genomic rearrangements and deletions after small-scale gene duplication processes in Lepidoptera could have resulted in the loss of a large number of dispensable genes (Albalat and Cañestro 2016). This would result in a higher rate for gene loss compared with gene gain as shown by our analyses (μ = 0.0032>λ = 0.0015).

### Gene Family Expansions and Contractions in Four Lepidopteran Families

We further focused on the expansion rates within four lepidopteran families. The cutworm moths (Noctuidae) are a large cosmopolitan and species rich radiation of moths of which many species are major polyphagous herbivores (van Nieukerken et al. 2011; Regier et al. 2017). Numerous major polyphagous species, such as those of the genus *Spodoptera*, are considered notorious pests causing significant agricultural damage worldwide (Pogue 2002; Cho et al. 2008; Sharanabasappa et al. 2018; Stacke et al. 2018). All Noctuid species included in our data set are reported pests and all except of the maize stalk borer, *Busseola fusca*, have a polyphagous feeding habit (fig. 1 and supplementary table 11, Supplementary Material online). The other families included in our comparison (Papilionidae, Nymphalidae, and Pieridae) primarily consist of monophagous species without a pest status (fig. 1). The majority of the species within these three families feed on a narrow host plant range, as indicated by the low PD and FMD values (supplementary table 11, Supplementary Material online and fig. 2). For Papilionidae only 21% of the 281 species have a polyphagous feeding habit, accepting more than one plant family (Scriber et al. 1991), whereas the Pieridae primarily feed on a restricted range of plants within three Angiosperm orders: Fabales, Santalales, and Brassicales (Braby and Trueman 2006). Within Nymphalidae, major polyphagous species do occur, but most species have a limited host plant range (Nylin et al. 2014; de la Paz Celorio-Mancera et al. 2016).

The Nymphalidae show high dynamic genome evolution rates when looking at rates of gene gains and losses, as calculated using all different data sets, in comparison to the other families (fig. 4). This is consistent with Nylin et al. (2014) who found that polyphagy in Nymphalidae was transient and that selection favored the host plant specialization with similar specialized metabolites. This oscillation of host plant ranges, termed the “oscillation hypothesis,” may contribute to phytophagous insect diversification when ancestral specialists give rise to plastic generalists that in turn adapt, diversify, and again specialize (Janz et al. 2006; Janz and Nylin 2008). When host specialization is currently selected for and becomes more dominant in Nymphalidae, this may be associated with the higher rate for gene family contraction (fig. 4).

We specifically looked at five gene families involved in detoxification of specialized metabolites (Schuler 2011; Heidel-Fischer and Vogel 2015; Kant et al. 2015), and compared the expansion and contraction rates, calculated using the “5 gene families data set,” across the lepidopteran families. In contrast to Nymphalidae, the Noctuidae and Pieridae both had a higher rate for gene gain (fig. 4*B*). The overall PD, and FMD values of noctuids indicate a wider range of accepted plant families in comparison to the other lepidopteran families (PD [1–22.04] and FMD [0.13–0.71], fig. 2). The higher expansion rates of detoxification gene families in Noctuidae suggest a correlation between their expansion and the evolution of polyphagy. However, the higher expansion rate in monophagous Pieridae might indicate that expansions are not exclusive to major polyphagous lineages. The lower PD (1–1.16) and FMD (0.03) values in Pieridae (and the overall high occurrence of monophagous species) does not explain the higher rate for gene family expansion given their restricted host ranges (Braby and Trueman 2006). Indeed, gene duplications occur in all organisms and can result in selective advantages due to subfunctionalization and/or neofunctionalization (He and Zhang 2005; Rastogi and Liberles 2005; Heidel-Fischer et al. 2019). For example, in Pierinae (a subfamily within Pieridae), gene duplication followed by neofunctionalization resulted in the evolution of the nitrile-specifier protein involved in the detoxification of glucosinolates produced by Brassicaceae plants (Wittstock et al. 2004; Wheat et al. 2007; Fischer et al. 2008).

Further, we looked at the rate of change (λ) of individual gene families involved in detoxification and digestion, calculated using the “single gene family data sets” (fig. 4*C*). Besides the selected five detoxification families, we added the trypsin and insect cuticle protein gene families. Trypsin, a family of serine proteases is involved in the hydrolyses of proteins and plays a role in the digestion of plant material in herbivorous invertebrates (Rawlings and Barrett 1994; Muhlia-Almazán et al. 2008). The insect cuticle protein family, involved in formation of the exoskeleton, is suggested to play a role in increased protection of the peritrophic matrix and midgut, forming a physical barrier for biochemical toxins (Hegedus et al. 2009; Agrawal et al. 2014; Kelkenberg et al. 2015; Kumar et al. 2018). The distribution of the calculated λs between the Lepidoptera families is similar for all gene families, with Nymphalidae showing the highest rates (average = 0.0073) followed by either Noctuidae (average = 0.0038) or Pieridae (average = 0.0034) and Papilionidae (average = 0.0016). This may indicate that selection forces act similar on gene families involved in plant feeding within Lepidoptera families. However, the differences in rate of change were not in proportion or equal to the level of polyphagy when comparing the PD and FMD values (fig. 2). For example, the Noctuidae that include species with the highest level of polyphagy (PD ranging between 1–22.04 and FMD between 0.13 and 0.71) did not correspond with highest rates of change, λ. This might indicate that diet breadth is not the dominant factor contributing to high gene turnover rates in these seven gene families and that other factors, like oscillating host ranges, are equally important.

### Gene Family Expansions and Diet Breadth

We analyzed gene family expansions of the seven gene families involved in plant feeding and diet breadth to test the correlation between gene family size and level of polyphagy. We observed size differences of gene families across the species of the four focal lepidopteran families (fig. 2). Indeed, Noctuidae have the widest range of accepted host plant families (PD [1–22.04] and FMD [0.13–0.71], fig. 2), and holds the highest number of genes for all the compared gene families (table 1). Within Noctuidae, *Spodoptera* (*S. litura* and *S. frugiperda*) had the highest overall gene count (table 1). Looking at the range of accepted plant families, *Spodoptera* has the largest host family range of all tested species (*S. frugiperda*: 74 families, PD = 22.04, FMD = 0.71; *S. litura*: 28 families, PD = 10.38, FMD = 0.34; *Spodoptera exigua*: 35 families, PD = 11.07, FMD = 0.39; fig. 2, supplementary table 11, Supplementary Material online). Genome analyses of these species (Cheng et al. 2017; Gouin et al. 2017; Gui et al. 2020; Xiao et al. 2020), and this study showed expansions in gene families involved in detoxification (fig. 2, table 1, and supplementary table 4, Supplementary Material online). These expansions are in line with the large breadth of host plant families and might enable the level of polyphagy. In contrast, the cabbage looper (*Trichoplusia ni*), another major polyphagous species (33 families, PD = 10.79, FMD = 0.35) has lower gene counts for all detoxification families (fig. 2, table 1, and supplementary table 4, Supplementary Material online), indicating that expansion levels vary among major polyphagous Noctuidae.

Further, whereas the range of the seven gene family sizes was relatively similar in Papilionidae and Pieridae, there were larger differences in family sizes across Nymphalidae (fig. 2). The majority of the included nymphalids are monophagous (stable PD and FMD values), whereas gene family sizes are highly variable (fig. 2 and supplementary table 4, Supplementary Material online). This indicates that expansions in gene families involved in plant feeding are not restricted to polyphagous species. For example, the meadow brown butterfly (*M. jurtina*), which showed the largest number of total gene counts (table 1), is found in grasslands, open woodland areas, and forest- and field-edges throughout the Palearctic region and is specializing on grasses (Poaceae), and thus considered a monophagous species (Tshikolovets 2011; Singh et al. 2020) (supplementary table 11, Supplementary Material online).

### Testing the Correlation between Gene Family Expansions and Polyphagy

Previous studies have reported major gene family expansions associated with polyphagy in individual species, such as *ABC* genes in the spider mite, *Tetranychus urticae* (Dermauw et al. 2013; Dermauw and Van Leeuwen 2014), *GST* genes in the red flour beetle, *Tribolium castaneum* (Shi et al. 2012), and *P450* genes in the fall armyworm, *S. frugiperda* (Gouin et al. 2017; Gui et al. 2020; Xiao et al. 2020). Here, we found a significant positive correlation between the gene family sizes of the detoxification gene families *CCE* and *GST* and level of polyphagy (fig. 3). *CCE*s are involved in the first phase of specialized plant metabolite detoxification by modifying the metabolite through hydrolysis (Oakeshott et al. 2005; Montella et al. 2012). This activates the compound for the second detoxification phase involving *GST*s that catalyze the conjugation of the tripeptide L-glutathione (GSH) and electrophiles, which increases the solubility of the compound and thus increases the ease of excretion (Armstrong 1997; Francis et al. 2005; Shi et al. 2012). The significant positive correlation indicates that a higher gene count of the *CCE* and *GST* families may increase the flexibility and range of host plant families by detoxification of a wider range of metabolites in polyphagous Lepidoptera. We did not find a significant correlation for the other gene families. This is in contrast to an earlier comparison of seven lepidopteran genomes where a correlation was found for subfamilies within clan 3 of the *P450* gene family and host plant breadth (Calla et al. 2017). We focused on complete gene families and therefore, we acknowledge that gene members might be included in the gene family counts that are not involved in detoxification. It is important to note that the study of Calla et al. (2017) indicate that within gene families expansion/loss rates might differ between smaller groups of genes (such as subfamilies within clan 3) which could correlate with host plant breadth. This could also lead to the different outcome of the studies.

Our data show that putative expansions of gene families involved in plant feeding are species-specific and not restricted to (major) polyphagous species alone. A significant correlation is only found for the *CCE* and *GST* gene families in polyphagous Lepidoptera. Expansion in these families is correlated with an increased level of polyphagy and may enable increased levels of polyphagy.

## Conclusions

Using available whole-genome data, we studied the association between polyphagy and gene family expansions across Lepidoptera. For each species, we calculated the PD and specialized metabolite content (FMD) of the host plants within each butterfly/moth diet to quantify level of polyphagy. Expansions of gene families involved in plant feeding were found in both monophagous and polyphagous species. Evolutionary expansion rates varied across Lepidoptera families, but were not proportionally higher in the Noctuidae, a lepidopteran family with widest host plant ranges (highest PD and FMD values). However, we observed a significant positive correlation between the gene expansion of *CCE* and *GST* detoxification families and host plant family range (PD and FMD values) across polyphagous Lepidoptera. We therefore conclude that expansions of gene families involved in plant feeding are species-specific and occur in both monophagous and polyphagous species, but particular gene families, *CCE* and *GST*, were positively correlated with level of polyphagy.

## Materials and Methods

### Data Sources and Quality Assessment

Annotation files and gene sets (protein translations) of 37 Lepidoptera genomes and one outgroup species (Trichoptera) were downloaded from various databases, including Ensemble LepBase release v. 4 (Challi et al. 2016) and NCBI (Sayers et al. 2020). The included species, data sources, and accession dates are reported in supplementary table 1, Supplementary Material online (All supplementary data are uploaded to the 4TU Centre for Research Data repository and available online: https://figshare.com/s/68b3db174aef43f9608f; reserved doi: 10.4121/16760824).

When genes were represented by multiple isoforms per gene (e.g., based on the sequence names), sequence files were edited using the Trinity based perl script “get_longest_isoform_seq” to ensure a single representative longest isoform. Completeness of genome gene sets were assessed using the *Insecta_odb9* gene set, consisting of 1,658 BUSCO in BUSCO v. 3.0.2. (Simão et al. 2015). BUSCO results showing high duplication levels in the gene set could indicate the presence of a high number of isoforms. In case high duplication levels were found, we checked the full genome assembly for the degree of gene duplication to see if high duplication levels actually reflected true duplications. In case a large difference between the protein set and genome assembly was shown, we assumed multiple isoforms per gene were still present and assessed CD-HIT-EST v. 4.8.1. (Li and Godzik 2006) using a 95% identity threshold. We applied CD-HIT-EST on *H. melpomene melpomene, H. erato demophoon, Leptidea sinapis*, and *Heliothis virescens*.

### Functional Annotation and Orthology Prediction

Peptide sequences were cleaned of diverse characters like “*” and “.” to avoid the use of illegal characters for the annotation analysis (e.g., InterProScan). We used InterProScan v. 5.36-75 (-appl Pfam—goterms) (Jones et al. 2014) for general annotation and identification of protein families. Further, we ran a local BlastP v. 2.6.0 (Camacho et al. 2009) against the UniRef50 database (https://uniprot.org/pub/databases/uniprot/uniref/uniref50/uniref50.fasta.gz; release version July 31, 2019, accessed August 20, 2019) (UniProt Consortium 2019) using a cut-off *e-*value of 1e-3. The annotated proteins using InterProScan and local BlastP were used to retrieve gene counts for the gene families of interest. Further, OrthoFinder v. 2.2.7 (Emms and Kelly 2015) was used to predict orthologous protein groups (OGs). An OG is a group of genes descended from a single gene in the last common ancestor of a group of species. The protein sequence files were used as input and OrthoFinder was run under default settings. We used the resulting orthologous protein groups as input for CAFE v. 4.2.1 (Hahn et al. 2005; De Bie et al. 2006). Since we focused on various gene families involved in plant feeding, we selected candidate OGs based on the BlastP and InterProScan identifications. We selected OGs of gene families of interest if genes matched one of the Uniref50 cluster terms, Pfam families or InterProScan identifiers specific for each gene family (supplementary table 5, Supplementary Material online). The gene families of interest were: P450 monooxygenases (*P450*s), *CCE*s, *UGT*s, *GST*s, *ABC*s, trypsin, and the insect cuticle protein family.

### Time-Calibrated Species Phylogeny

The CAFE analyses required an ultrametric phylogeny of the Lepidoptera. We used the protein sequences of single-copy BUSCO genes to generate alignments of orthologous genes. All 1,367 single-copy BUSCO proteins were individually retrieved for every species on the amino acid (aa) level using BUSCO v. 4.0.5., applying the *insecta_odb10* as a reference lineage data set (Simão et al. 2015). Individual alignments were generated for every BUSCO-identified ortholog using MAFFT v. 7.305 (Katoh and Standley 2013) using the L-INS-i algorithm. For the identification of putative ambiguously aligned or randomized multiple sequence alignment (MSA) sections, we used Aliscore v. 1.2 (Misof and Misof 2009; Kück et al. 2010) on each MSA with the default sliding window size, the maximal number of pairwise sequence comparisons, and a special scoring for gap-rich amino acid data (options -r and -e). After exclusion of the identified putative ambiguously aligned or randomized MSA sections with ALICUT v. 2.3 (Kück et al. 2010), the final MSAs were concatenated into a supermatrix using FASconCAT-G v. 1.02 (Kück and Longo 2014). The resulting data set comprised 1,367 gene partitions and 687,494 amino acid positions.

Prior to the tree reconstruction, the best scoring amino acid substitution matrix for each gene partition was selected with ModelFinder as implemented in IQ-TREE v. 1.6.12 (Kalyaanamoorthy et al. 2017). We restricted the search of the best fitting model to eight amino acid substitution matrices appropriate for nuclear markers: DCMut (Kosiol and Goldman 2005), JTT (Jones et al. 1992), LG (Le and Gascuel 2008), Poisson, PMB (Veerassamy et al. 2003), VT (Muller and Vingron 2000), and WAG (Whelan and Goldman 2001). We additionally included the protein mixture model LG4X (Le et al. 2012), which accounts for FreeRate heterogeneity. Furthermore, we allowed testing the default rate heterogeneity types (E, I, G, I + G, and FreeRates: R) (Yang 1994; Gu et al. 1995; Soubrier et al. 2012), with or without empirical rates (-F, -FU) as well as testing the number of rate categories (-cmin 4 -cmax 15). The best model for each gene partition was selected according to the best second-order or corrected Akaike Information Criterion (AICc) score (Hurvich and Tsai 1989). Data set and partition scheme including selected models can be found at the 4TU Centre for Research Data repository available online: https://figshare.com/s/68b3db174aef43f9608f (reserved doi: 10.4121/16760824).

Phylogenetic relationships were inferred under the ML optimality criterion as implemented in IQ-TREE v. 1.6.12 (Nguyen et al. 2015; Chernomor et al. 2016) using the best scoring amino acid substitution matrix for each gene partition and the edge-proportional partition model allowing partitions to have different evolutionary rates (option -ssp). We performed 50 independent tree searches (25 searches with a random and 25 with a parsimony start tree). The resulting number of unique tree topologies was assessed with Unique Tree v. 1.9, kindly provided by Thomas Wong and available upon request. We used the ML tree with the best log-likelihood to obtain an ultrametric tree using the chronos function of the R package ape v. 5.4 on R v. 3.6.3, relaxed model (Paradis et al. 2004; R Development Core Team 2020). The tip to root length was adjusted to match the approximately 299.5-Myr evolutionary history of crown group Lepidoptera (Kawahara et al. 2019).

### Ecological Host Data and Diversity Metrics

For each lepidopteran species, we collected host plant specialization level, host plant family and species range, pest status, and specialized metabolite content within the accepted host plant range. Data were collected by browsing literature for host plant species accepted by each of our butterflies and moths studied. We used this information to determine for each lepidopteran species the range of host plant acceptance, and subsequently classified diet breadth (level of polyphagy or monophagy; supplementary table 11, Supplementary Material online). Additionally, we recorded pest status of the lepidopteran species if the species was a described pest in the literature searched or if included in the EPPO or CABI databases (EPPO Global Pest Database 2019; https://gd.eppo.int; CABI ISC 2020). We considered all lepidopteran species that accept host plant species from a single plant family to be a monophagous species. Species recorded as polyphagous were those feeding on species from multiple plant families.

To quantify the PD of a particular lepidopteran species’ diet, we calculated the Faith’s measure of PD (Faith 1992). To calculate the PD for each range of host plant families, we used the package Picante v. 1.8.2 (Kembel et al. 2010) in R v.3.6.2 (R Development Core Team 2020). This metric quantifies the degree of host plant range diversity by calculating the distance between plant families according to branch lengths of a reference phylogeny. As a reference phylogeny, we used the recent angiosperm phylogeny of Ramírez-Barahona et al. (2020), pruned for lepidopteran host plant families. Calculated PD values were scaled so that monophagous species had a PD = 1 (all PDs divided by 374.14, the value for single gene family acceptance). Two families included in our list of recorded host plant families, Aspleniaceae and Araucariaceae, were missing in the reference phylogeny. These plant families are hosts for only two highly polyphagous species in our analysis (*S. frugiperda* [Aspleniaceae] and *S. exigua* [Araucariaceae]). Thus, we expected that their exclusion would have a very small impact on the dietary PD. Accordingly, we removed Aspleniaceae from our data set, whereas we replaced Araucariaceae by Cupressaceae, the next most closely related family in our reference phylogeny.

We compiled reported specialized metabolites for each host plant family. We collected information for the three main groups of secondary metabolites, as classified in Schoonhoven et al. (2005): phenolics, terpenoids, and nitrogen-containing compounds. For each host plant family, we recorded the secondary metabolite type, chemical class, subclass and, if present, any additional sublevel (supplementary table 13, Supplementary Material online). Metabolites belonging to the same chemical type or class are by definition more similar. Thus, we used a hierarchical structure to calculate the degree of FMD of specialized metabolites encountered by the lepidopteran species in their range of accepted host plant families. Plant families with similar chemical compositions are likely to be detoxified by similar mechanisms. Lepidopteran species feeding on plants with diverse specialized metabolites will thus have a higher value for the FMD (e.g., polyphagous species).

We used the database of specialized metabolite records per plant family to create a trait matrix (supplementary table 17, Supplementary Material online), the first step to calculate a functional diversity index (Petchey and Gaston 2002, 2006). Afterwards, we calculated the dietary FMD of each lepidopteran species with a dendrogram-based method using the script by Schumacher J and Petchey OL (accessed February, 2021; http://github.com/opetchey/dumping_ground/tree/master/functional_diversity/FD.example.2) as described in Petchey and Gaston (2002). As a consequence of the dendrogram-based calculation method, the FMD could only be calculated for polyphagous species because of the range of accepted metabolites. Measures of PD and FMD could not be calculated for the Indian meal moth, *Plodia interpunctella*, because this species feeds exclusively on dried products such as stored and processed food, and thus the influence of specialized metabolites is limited.

We calculated a Spearman rank correlation coefficient to examine the correlation between degree of polyphagy, using the PD and FMD metrics, and gene counts of gene families involved in plant feeding. Specifically, we used the gene counts of plant detoxification related gene families (*P450*, *CCE*, *UGT*, *GST*, and *ABC*) and the trypsin and insect cuticle protein families. Correlation analyses of gene family counts (supplementary table 4, Supplementary Material online) and both PD and FMD (supplementary tables 12 and 14, Supplementary Material online) were analyzed. Correlation statistics were calculated using the function “cor.test” in the package Stats v. 3.6.2 in R v. 3.6.2 (R Development Core Team 2020).

*Spodoptera frugiperda* is represented in our data set by both the rice and the corn strain, belonging to the same species. Therefore, we additionally tested the correlation significance when only a single *S. frugiperda* strain (rice population, with the lowest gene counts) was included.

### CAFE Analysis

We used CAFE v. 4.2.1 (Hahn et al. 2005; De Bie et al. 2006) to analyze gene family evolution (gene gains and losses) in a phylogenetic context. CAFE uses a birth and death process to model gene gain and loss across an ultrametric phylogenetic tree. Based on the results of OrthoFinder, gene counts per species were used as input for the CAFE analyses.

Gene families that have large variance in gene copy numbers across species can cause the parameter calculations to be noninformative (CAFE tutorial documentation v. 20 January 2016). From a computational perspective filtering out high variance OGs is needed in order to let the statistical analyses reach saturation. Therefore, the gene count data set as derived from the OrthoFinder run was filtered for OGs with high variance levels. We filtered out all OGs which showed ≥100 copies, as recommended by the developers (CAFE tutorial documentation v. 20 January 2016) in at least one species. After testing if CAFE reached convergence with multiple thresholds, we finally removed the top 2% OGs with highest variance. This resulted into the data set including OGs comprising all gene families, called hereafter the “all gene families data set.” Based on this data set, we calculated the error model because errors in genome assembly and gene annotation potentially result in biased evolutionary rate estimates (Han et al. 2013). We used *caferror.py*, as provided in CAFE, to calculate the error in our data set due to assembly and annotation mistakes. The method accounts for errors by modeling the uncertainty of observed family sizes at the tips of the tree (Han et al. 2013). The resulting model based on the “all gene families data set” was used in all CAFE runs analyzing the data sets as described below.

We generated one additional data set which was filtered for OGs belonging to five specific gene families involved in specialized metabolite detoxification: *P450*s, *CCE*s, *UGT*s, *GST*s, and *ABC*s, called hereafter “5 gene families data set.” In addition, we applied CAFE on selected single gene family data sets to study rates of change per gene family. These selected single gene families were the five detoxification families (*P450*, *CCE*, *UGT*, *GST*, and *ABC*) and two additional families potentially important for (polyphagous) herbivory: the insect cuticle protein family and the trypsin gene family. These data sets are called hereafter “single gene family data sets.”

The CAFE runs included the calculation of the single rate of change (λ), and a second mode where gains (λ) and losses (μ) were calculated separately. The *P* value threshold was kept at the default value (0.01), and the top 2% high variance OGs were removed in all data sets in order to let CAFE reach saturation. Multiple runs of CAFE were used to test for convergence. To reach convergence, only a single rate of change (λ) was calculated for the “single gene family data sets,” whereas both gain- (λ) and loss- (μ) rates were calculated for the “all gene families data set” and “5 gene families data set.” In all the analyses, the trichopteran outgroup, *Limnephilus lunatus*, was treated as a separate group calculating an individual λ and μ. We similarly treated *M. jurtina*, the meadow brown, as a separate group within Nymphalidae because it had a higher number of predicted genes than the other species (*M. jurtina*: 36,294, Nymphalidae average excluding *M. jurtina*: 17,554) and thus expected to have a different rate of change. By treating *M. jurtina* as separate group, we avoid the potential overestimation of the rates of change due to the higher number of predicted genes in comparison to the other species.

Single expansion and contraction rates based on the entire phylogeny were only calculated for the “all gene families data set.” In addition, we also ran several analyses calculating separate rates of change for specific clades in the tree to address the evolution of polyphagous herbivory. Specifically, we analyzed four target lepidopteran families for which more than two species were included in the data set: cutworm moths (Noctuidae), swallowtails (Papilionidae), brush-footed butterflies (Nymphalidae), and whites (Pieridae). For these four families, expansion and contraction rates were calculated using the “all gene families data set,” “5 gene families data set,” and the “single gene family data sets.” 

## Supplementary Material

Supplementary data are available at *Genome Biology and Evolution* online.

## Author Contributions

T.B. and S.S. designed the study. Initial data collection, processing, and analyses by T.B. Further analyses, and discussions on the results and data were done by T.B., S.S., and M.E.S. C.F.H.L. collected the plant and metabolite data and constructed the host plant and metabolite databases, and calculated the PD and FMD metrices. The correlation statistics was done by C.F.H.L. and T.B. The manuscript was written by T.B. and S.S., with further revisions based on comments and discussions from M.E.S., and input from C.F.H.L. and V.I.D.R. V.I.D.R. provided the *Spodopteraexigua* data. All authors read and approved the final manuscript.

## Data Availability

The data underlying this article are available in the article and in its Supplementary Material online found at the 4TU Centre for Research Data repository available online DOI: 10.4121/ 16760824.

## Supplementary Material

evab283_Supplementary_Data
