# Corrigendum to: Expanding the Menu: Are Polyphagy and Gene Family Expansions Linked across Lepidoptera?

**DOI:** 10.1093/gbe/evac029

**Published:** 2022-02-25

**Authors:** Thijmen Breeschoten, Corné F H van der Linden, Vera I D Ros, M Eric Schranz, Sabrina Simon

Genome Biology and Evolution, Volume 14, Issue 1, January 2022, evab283, https://doi.org/10.1093/gbe/evab283

In the originally published version of this manuscript, the first author’s name is incorrectly missing from the following citation:

Kalleshwaraswamy C, et al. 2018. First report of the fall armyworm, Spodoptera frugiperda (Lepidoptera: Noctuidae), an alien invasive pest on maize in India. Pest Manag Horticult Ecosyst. 24:23–29.

The correct citation should be as follows; this has been updated:

Sharanabasappa D, et al. 2018. First report of the Fall armyworm, Spodoptera frugiperda (JE Smith) (Lepidoptera: Noctuidae), an alien invasive pest onmaize in India. Pest Manag Horticult Ecosyst. 24:23–29.

