# Supplementary material for: Expanding the Menu: Are Polyphagy and Gene Family Expansions Linked across Lepidoptera?
Source: Genome Biol Evol. 2021 Dec 24;14(1):evab283. doi: 10.1093/gbe/evab283 (PMC8725640; doi:10.1093/gbe/evab283)
Supplement: evab283_Supplementary_Data [file evab283_supplementary_data.zip › Supplementary datafiles.docx]

**Supplementary datafiles**

All Supplementary Data is uploaded to the 4TU Centre for Research Data repository and available online: https://figshare.com/s/68b3db174aef43f9608f (reserved DOI: 10.4121/16760824)

Supplementary Table 1. Overview of genomes including source location and accession dates.

Supplementary Table 2 (A-C). InterProScan annotation report for all 38 specimens.

Supplementary Table 3 (A-C). Annotation report of the proteins from all 38 specimens of the BLASTP run against the UniRef50 reference database.

Supplementary Table 4. Number of gene members annotated using InterProScan and a local BLASTP against the UniRef50 protein reference database for the five main detoxification gene families P450 monooxygenases (P450s), carboxyl- and choline esterases (CCEs), UDP-glycosyltransferases (UGTs), glutathione S-transferases (GSTs) and ATP-binding cassettes (ABCs) and the gene families trypsin and insect cuticle proteins.

Supplementary Table 5. Gene family identifiers using InterProScan identifiers and UniRef cluster terms. Based on these identifiers, putative gene members from gene families P450, CCE, UGT, GST, ABC, trypsins and insect cuticle proteins were selected for the orthogroup candidates.

Supplementary Table 6. Orthogroups identified using OrthoFinder v. 2.2.7 including translated proteins from all 37 Lepidoptera, and single Trichoptera species as included in this study.

Supplementary Table 7. Gene counts per orthogroup of all orthogroups identified
using OrthoFinder.

Supplementary Table 8. *All gene families dataset* as used in the CAFE analyses analysing all gene families. This dataset is filtered for high variance OGs, and also used to calculate the error model.

Supplementary Table 9. *5 gene families dataset* as used in the CAFE analyses to analyse gene evolution of the 5 selected detoxification gene families (P450, CCE, UGT, GST and ABC).

Supplementary Table 10. *Single gene family datasets* as used in the CAFE analyses of the seven selected gene families involved in plant feeding (P450, CCE, UGT, GST, ABC, trypsin and insect cuticle protein).

Supplementary Table 11. Overview of host plant range, feeding style and pest status for all species included in this study.

Supplementary Table 12. Faith's phylogenetic diversity index (PD) per lepidopteran species based on the accepted host plant family range.

Supplementary Table 13. Overview of specialized metabolite content per host plant family. For each family the metabolite content on metabolite type, class, subclass and sub-level is provided.

Supplementary Table 14. Functional metabolite diversity index (FMD) per lepidopteran species based on metabolite content for host plant family range.

Supplementary Table 15. Overview of correlation and significance tests between level of polyphagy (quantified by PD and FMD index values) and gene counts of various gene families (P450, CCE, UGT, GST, ABC, trypsin and insect cuticle protein) in polyphagous lepidopteran species.

Supplementary Table 16. Overview of CAFE results for the analyses of gene family evolution in terms of gene gain and loss rates.

Supplementary Table 17. Trait matrix based on the database of specialized metabolite records per plant family as used to calculate the functional metabolite diversity index (FMD).
